# Lifetime and twelve-month prevalence of heavy-drinking in Singapore: Results from a representative cross-sectional study

**DOI:** 10.1186/1471-2458-13-992

**Published:** 2013-10-21

**Authors:** Wei-Yen Lim, Mythily Subramaniam, Edimansyah Abdin, Vincent Yaofeng He, Janhavi Vaingankar, Siow Ann Chong

**Affiliations:** 1 Saw Swee Hock School of Public Health, National University of Singapore and National University Health System, MD3, 16 Medical Drive, S(117597), Singapore, Singapore; 2 Research Division, Institute of Mental Health, Singapore, Singapore

**Keywords:** Heavy drinking, Alcohol abuse, Quality of life, Psychiatric co-morbidity

## Abstract

**Background:**

The study aimed to establish the prevalence of heavy drinking, evaluate correlations between heavy drinking and socio-demographic factors, physical and psychiatric conditions, and assess the impact of heavy drinking on quality of life and days of work-loss.

**Methods:**

Data from a nationally-representative cross-sectional sample were used. The sample comprised 6616 community-dwelling Singaporeans & Singapore Permanent Residents. The main instruments used were the World Mental Health Composite International Diagnostic Interview and EuroQol 5D. Heavy drinking was defined as consumption of 4 or more drinks, or 5 or more drinks in a day in women and men respectively.

**Results:**

12.6% of all adult Singapore residents reported heavy drinking in the last 12 months, and 15.9% reported lifetime heavy-drinking. Strong gender, ethnic, age and income differences were seen. Heavy drinking was positively associated with major depression, the presence of any mood disorder, and with chronic pain. It was also strongly associated with alcohol dependence, alcohol abuse, and nicotine dependence. Heavy-drinkers reported lower quality of life compared to non-heavy drinkers, measured using the EuroQol 5D Visual Analogue Scale.

**Conclusions:**

Singapore has a relatively high prevalence of 12-month heavy drinking of 12.6%, and lifetime heavy drinking of 15.9%. Heavy drinking was positively associated with both physical and mental health conditions, and with declines in quality of life. Continued monitoring of heavy drinking behavior and sustained efforts to mitigate the risks associated with heavy drinking is needed.

## Background

Adverse effects of over-consumption of alcohol are well-documented, and two forms of alcohol use disorder- alcohol abuse and alcohol dependence, are classified in the Diagnostic and Statistical Manual of Mental Disorders, 4^th^ edition (DSM-IV) of the American Psychiatric Association. However, some patterns of alcohol consumption may be harmful even in the absence of alcohol abuse or dependence, and binge drinking is recognised as a potential cause of morbidity and mortality [1-7].

Binge drinking refers to high volume alcohol consumption over a short period of time, and is frequently defined as the consumption of four or more drinks at a single sitting for women, and of five or more drinks at a single sitting for men [8]. Binge drinking is associated with physiological effects such as behavioural disinhibition, temporary impairment of judgment, nausea and vomiting, and hangover symptoms. Studies have linked binge drinking to increased coronary heart disease risk [1], psychiatric morbidity [2], and cognitive impairment [3]. Children born to women who binge drink during the pregnancy appear to have a higher risk of psychiatric and behavioural difficulties [4]. In addition, binge drinking is also associated with a myriad of social issues–and is associated with risky sexual behavior [5,6], violence [5], and injuries resulting from motor and other accidents [5,7]. Overall, binge drinking is relatively prevalent; data from representative samples in the US suggest a 30-day period prevalence of 17.1% [9], while data from Brazil suggest that 26.7% had at least 1 episode of binge-drinking in the past year [10].

Singapore is an island city-state in South-East Asia, with a multi-ethnic population of about 5 million, with 3.77 million Singapore citizens and Permanent Resident [11]. The resident population comprises Chinese (74.1%), Malays (13.4%), Indians (9.2%) and Other ethnic groups (3.3%) (11). We recently showed that the prevalence of alcohol use disorders as established using DSM-IV was relatively low in Singapore, with a lifetime prevalence of alcohol abuse and alcohol dependence of 3.1 and 0.5% respectively [12]. We had also earlier noted an increase in past-month binge drinking rates in the Singapore resident population, from an age-adjusted prevalence of 5.1% in 1992 to 10% in 2004 [13], based on data obtained from the National Health Surveys, which were representatively-sampled serial cross-sectional surveys conducted in Singapore with a 6-yearly interval. The definition of binge drinking was operationalised as consuming 5 drinks or more in a single drinking session for both men and women. The data available suggest that while diagnoses of alcohol use disorders were low, harmful patterns of alcohol drinking may be relatively common in Singapore.

In this paper, we examine the prevalence of heavy drinking (as a proxy for binge drinking) using data from a nationally representative cross-sectional survey. We also evaluated correlations between heavy drinking and socio-demographic factors and the concomitant presence of self-reported physical conditions and psychiatric conditions (based on DSM-IV criteria). Finally, we assessed the impact of heavy drinking by evaluating differences in self-reported quality of life and days of work-loss.

## Methods

### Study

Data from the Singapore Mental Health Study (SMHS) were used. The design and conduct of this study have been previously described [14]. Briefly, the SMHS was a representative cross-sectional survey of adult Singapore residents conducted from December 2009 to December 2010. The population of interest were Singapore residents aged 18 years or older living in households and able to speak English, Mandarin or Malay. Participants were randomly selected from a sampling frame of all household addresses in Singapore. Exclusion criteria were foreigners residing in Singapore, persons younger than 18 years of age, persons unable to complete an interview due to severe physical or mental conditions or language barriers, persons who were institutionalised or hospitalised at the time of the survey, and persons who were uncontactable. A disproportionate stratified sampling was used with oversampling of older people, and equal proportion sampling of the three main ethnic groups of Chinese, Malays and Indians. Sampling weights were then used to adjust for the oversampling. Results were then further adjusted for age, ethnicity and gender to the Singapore resident population based on the Department of Statistics estimates for the year 2007.

Ethical approval was obtained from the Domain-Specific Review Board of the National Healthcare Group in Singapore prior to implementation of the study, and full informed consent was obtained from participants and parents/guardians of participants aged 18–20 years. A participant’s age and ethnicity were obtained from the National Registration Identity Card. Other socio-demographic information were self-reported, including marital status, highest educational level achieved, household income, type of current residence and occupational status. Data were collected as categorical responses.

The diagnoses of mental disorders were based on the World Mental Health Composite International Diagnostic Interview version 3.0 (CIDI 3.0) [15]. All interviews were conducted by trained lay-interviewers. (CIDI 3.0 was developed specifically to be used by trained lay-interviewers). Modules for mood disorders, anxiety disorders (generalized anxiety disorder and obsessive compulsive disorder) and alcohol use disorders were included in the survey. Lifetime diagnoses of mental disorders were used for this analysis. Nicotine dependence was established using the 6-item Fagerstrom Test for Nicotine Dependence [16].

The question of the CIDI 3.0 module on alcohol use: “During the last 12 months, what was the largest number of drinks you had had in one single day?” was used to determine heavy drinking. Men who answered 5 or more drinks a day and women who answered 4 or more drinks were considered to have a history of heavy drinking in the last 12 months. A drink was defined as a glass of wine, a can or bottle of beer or a shot or jigger of liquor either alone or in a mixed drink.

All participants with history of heavy-drinking in the last 12 months were considered heavy drinkers over the lifetime. In addition, among those who were not heavy drinkers in the last 12 months, we used answers to the question: “Was there ever a year in your life when you drank more than you did in the past 12 months?” and: “On the days you drank during those years, about how many drinks did you usually have per day?”. Participants who reported that there was ever a year in their life where they drank more than they did in the last 12 months and who then reported 5 or more drinks a day (for men) or 4 or more drinks a day (for women) were considered heavy drinkers over the lifetime. We used this definition of heavy drinking as a proxy for binge drinking, since our questionnaire did not ascertain drinking volume over a single occasion.

Participants were also asked if their doctor had informed them that they had any of 15 specific medical conditions which were relatively prevalent in Singapore. These were: a) high blood sugar/diabetes, b) high blood pressure/hypertension, c) chronic pain (arthritis or rheumatism, back problems including disk or spine, and migraine headaches), d) cancer diagnosed in the last 3 years, e) neurological conditions (epilepsy, fainting spells or Parkinson’s disease), f) cardiovascular disorders (stroke or major paralysis, heart attack, coronary heart disease, angina, congestive heart failure or other heart disease), g) ulcers and chronic inflamed bowel diseases (stomach ulcer, inflamed bowel enteritis or colitis) and h) respiratory diseases (asthma, chronic lung diseases such as chronic bronchitis or emphysema).

An index was created using health states defined by the EuroQoL 5D instrument [17] to assess quality of life. This index was based on the utility of EuroQoL 5D health states elicited using the time trade-off method on a representative sample of the United Kingdom general population [18]. In addition, respondents were asked to evaluate their current health state along a Visual Analogue Scale (VAS), where 0 was the worst imaginable health state, and 100, the best imaginable health state. Self-reported loss in work days was elicited from the question in the 30-day functioning and disability module of the CIDI 3.0 instrument: “Beginning yesterday, and going back 30 days, how many days out of the past 30 were you totally unable to work or carry out your normal activities because of problems with your physical health, your mental health or your use of alcohol or drugs?”

### Statistical methods

All data analyses were performed using weighted data. Mean and standard deviations were calculated for continuous variables, and frequencies and percentages for categorical variables. Logistic regression was used to assess the association between heavy drinking and socio-demographic variables, mental illnesses and physical disorders. Linear regression was used to assess the association between heavy drinking and work days lost and health-related quality of life as measured using the EuroQoL 5D index and the EuroQoL VAS in the EuroQoL 5D instrument. To account for the effects of complex sample design due to stratification and weighting, standard errors and significance tests were estimated using the Taylor series linearization method. Multivariate significance was evaluated using the Wald test based on design corrected coefficient variance-covariance matrices. Statistical significance was set at the conventional level of p < 0.05, using two-sided tests. Statistical analyses were carried out using the statistical packages Stata version 10.0 (College Station, TX, USA) and the Statistical Analysis Software (SAS) system version 9.2 (Cary, NC, USA).

## Results

From an initial study frame of 13,500 individuals, 9116 (67.5%) were identified, and of these, 6648 (72.9%) were successfully interviewed. 854 (9.4%) refused to participate, 664 (7.3%) were either not contactable or were deceased, 554 (6.1%) had moved to another location or country, and 396 (4.3%) were ineligible due to language barrier or inability to complete the interview. Subsequently, 32 completed interviews were voided during the quality review, leaving a total study population of 6616 individuals. The overall response rate for the study, excluding the ineligible 396 cases, was 75.9% (n = 6616).

Participants in this study were almost equally divided between men and women. Malays and Indians were over-represented in this study to enable more stable and accurate ethnic-specific prevalence estimates of mental illnesses. 70% of the study population was younger than 50 years of age, and the majority of participants (81.6%) had at least secondary level education (Table 1). The weighted 12-month and lifetime prevalence of heavy drinking in the study sample were 12.6% (95% Confidence Interval (CI) 11.5%–13.7%) and 15.9% (95% CI 14.7%–17.1%) respectively (Table 1). The crude prevalence of 12-month and lifetime prevalence of heavy drinking was 11.3% and 14.3%, respectively.

**Table 1:** Association of lifetime and twelve-month prevalence of heavy-drinking with socio-demographic variables

		**Study pop (n)**	**Lifetime heavy drinking**				**Twelve-month heavy drinking**			
			**%**	**95% CI**	**Crude OR (95% CI)**^**1**^	**Adjusted OR (95% CI)**^**2**^	**%**	**95% CI**	**Crude OR (95% CI)**^**1**^	**Adjusted OR (95% CI)**^**2**^
Total		6616	15.9	14.7–17.1			12.6	11.5–13.7		
Gender	Men	3299	23.5	21.5–25.6	1.0	1.0	18.3	16.4–20.1	1.0	1.0
	Women	3317	8.7	7.4–10.0	**0.31 (0.25–0.38)**	**0.32 (0.25–0.40)**	7.4	6.2–8.6	**0.36 (0.29–0.44)**	**0.37 (0.29–0.48)**
Age group (Years)	18–34	2293	27.0	24.4–29.6	1.0	1.0	23.1	20.7–25.6	1.0	1.0
	35–49	2369	13.1	11.1–15.0	**0.41 (0.33–0.50)**	**0.33 (0.24–0.44)**	10.3	8.6–12.1	**0.38 (0.30–0.48)**	**0.36 (0.26–0.50)**
	50–64	1542	9.4	7.3–11.5	**0.28 (0.21–0.37)**	**0.25 (0.17–0.36)**	6.1	4.4–7.8	**0.22 (0.16–0.30)**	**0.24 (0.16–0.36)**
	65 and above	412	6.5	3.0–9.9	**0.19 (0.10–0.34)**	**0.20 (0.09–0.42)**	3.5	0.90–6.1	**0.12 (0.06–0.27)**	**0.19 (0.07–0.50)**
Ethnicity	Chinese	2006	16.0	14.5–17.6	1.0	1.0	12.7	11.4–14.1	1.0	1.0
	Malay	2373	6.3	5.3–7.2	**0.45 (0.37–0.54)**	**0.27 (0.22–0.34)**	3.9	3.1–4.6	**0.28 (0.22–0.35)**	**0.21 (0.16–0.28)**
	Indians	1969	15.4	13.8–17.0	0.90 (0.58–1.40)	0.78 (0.64–0.94)	12.5	11.1–14.0	0.98 (0.82–1.18)	0.83 (0.67–1.02)
	Others	268	61.3	55.1–67.4	**8.28 (6.24–11.00)**	**7.29 (5.00–10.67)**	54.7	48.4–61.1	**8.27 (6.22–11.00)**	**7.20 (4.93–10.51)**
Marital status	Single	1825	24.1	21.4–26.7	1.0	1.0	21.4	18.8–23.9	1.0	1.0
	Married	4290	12.4	11.0–13.8	**0.45 (0.37–0.54)**	0.81 (0.62–1.06)	9.0	7.6–10.2	**0.36 (0.29–0.45)**	**0.63 (0.47–0.84)**
	Divorced/Separated	262	22.2	15.1–29.4	0.90 (0.58–1.40)	**1.88 (1.12–3.16)**	17.7	11.3–24.1	0.79 (0.50–1.26)	1.62 (0.93–2.83)
	Widowed	237	5.5	0.3–10.7	**0.18 (0.07–0.51)**	0.97 (0.29–3.23)	2.4	−1.0–5.7	**0.09 (0.02–0.38)**	0.81 (0.18–3.72)
Annual income	Less than S$20 000	3392	12.8	11.1–14.4	1.0	1.0	9.9	8.4–11.3	1.0	1.0
	S$20000–S$49999	1924	18.3	15.9–20.7	**1.53 (1.22–1.91)**	1.16 (0.87–1.55)	14.6	12.3–16.8	**1.56 (1.22–1.99)**	1.10 (0.80–1.52)
	S$50000 and above	962	23.8	20.3–27.3	**2.14 (1.67–2.73)**	**1.75 (1.19–2.58)**	19.5	16.3–22.7	**2.21 (1.70–2.87)**	**1.67 (1.08–2.57)**
Education level	Completed primary school or lower	1236	7.1	4.9–9.3	1.0	1.0	3.9	2.3–5.6	1.0	1.0
	Secondary School	1975	13.2	10.9–15.4	**1.98 (1.34–2.91)**	1.33 (0.86–2.06)	10.0	8.0–12.0	**2.70 (1.65–4.43)**	**1.88 (1.07–3.33)**
	Pre-University/Junior College/Diploma/Vocational certificates	2063	20.4	17.9–22.9	**3.35 (2.32–4.84)**	1.13 (0.71–1.79)	17.0	14.7–19.3	**5.00 (3.12–8.00)**	1.66 (0.93–2.97)
	University	1342	21.2	18.3–24.0	**3.51 (2.41–5.11)**	0.89 (0.54–1.48)	18.0	15.3–20.6	**5.34 (3.32–8.60)**	1.35 (0.73–2.52)
Employment	Employed	4594	18.3	16.7–19.8	1.0	1.0	14.8	13.4–16.2	1.0	1.0
	Economically inactive^@^	1522	8.3	6.3–10.3	**0.40 (0.30–0.54)**	0.72 (0.48–1.06)	6.5	4.7–8.2	0.40 (0.29–0.55)	0.68 (0.44–1.06)
	Unemployed	313	20.1	13.4–26.8	1.13 (0.73–1.73)	1.37 (0.82–2.28)	13.6	8.1–19.0	0.90 (0.56–1.46)	1.00 (0.57–1.75)

@: includes homemakers, students and pensioners.

OR = Odds Ratios 95% CI = 95% Confidence Intervals. Estimates in bold are statistically significant.

^1^Weighted unadjusted bivariate analyses.

^2^Weighted adjusted multivariate analyses; adjusted for all other variables in the table.

The prevalence of heavy drinking differed significantly by socio-demographic variables. Men were more likely to drink heavily than women. Younger participants were more likely to drink heavily than older ones. Malays were less likely to drink heavily than the other ethnic groups, while those with an ethnicity of “Others” were more likely to drink heavily. Married and widowed individuals were less likely to drink heavily than single individuals. People with higher incomes were more likely to drink heavily. People of higher education status were more likely to drink heavily, while economically inactive people were less likely to drink heavily (Table 1).

After adjusting for socio-demographic factors, women were less likely to drink heavily than men (OR 0.37, 95% CI 0.29-0.48 for 12-month and OR 0.32, 95% CI 0.25-0.40 for lifetime prevalence), and Malays were less likely to drink heavily than Chinese (OR 0.21, 95% CI 0.16-0.28 for 12-month and OR 0.27, 95% CI 0.22-0.34 for lifetime prevalence), while those with an “Others” ethnicity were more likely to drink heavily (OR 7.20, 95% CI 4.93-10.51 for 12-month and OR 7.29, 95% CI 5.00-10.67 for lifetime prevalence). Older people were much less likely to drink heavily (OR 0.19, 95% CI 0.07-0.50 for 12-month and OR 0.20, 95% CI 0.09-0.42 for lifetime prevalence of heavy drinking in those 65 years and above, compared to those 18–34 years), while participants with high personal income were more likely to drink heavily (OR 1.67, 95% CI 1.08-2.57 for 12-month and OR 1.75, 95% CI 1.19-2.58 for lifetime prevalence in those earning S$50 000 and more annually, compared to those earning less than S$20 000 annually). Divorced or separated individuals were more likely to report lifetime heavy drinking compared to single persons (OR 1.88, 95% CI 1.12-3.16), although the increased OR for 12-month heavy drinking was not significant. Married persons were less likely to report 12-month heavy drinking (OR 0.63, 95% CI 0.47-0.84), although the decreased OR was not significant for lifetime heavy drinking (Table 1).

Among heavy drinkers, the prevalence of major depression was 6.2% over a 12-month timeframe, and 11.6% over a lifetime timeframe, while those in non- heavy drinkers were 1.6% and 4.7% respectively. This significant positive association between major depression and heavy drinking persisted after adjustment (OR 3.47, 95% CI 1.98-6.08 and OR 2.75, 95% CI 1.88-4.00 respectively). Bipolar disorder was more prevalent among lifetime heavy drinkers than non-heavy drinkers (2.5% vs 1.0%, adjusted OR 2.17, 95% CI 1.08-3.92), although there was no associations seen with 12-month heavy drinking. Collectively, mood disorders were associated with both 12-month and lifetime heavy drinking (OR 2.96, 95% CI 1.79-4.92, and OR 2.78, 95% CI 1.98-3.92 respectively). Obsessive-compulsive disorder and all anxiety disorders were associated with lifetime heavy drinking in bivariate analyses, but this association was no longer significant after adjustment. Alcohol abuse and overall alcohol use disorders were both significantly associated with 12-month and lifetime heavy drinking. The prevalence of nicotine dependence among 12-month heavy drinkers was 10.2%, and 9.6% among lifetime heavy drinkers, compared to non- heavy drinkers at 3.6 and 3.4% respectively. These associations remained significant after adjustment (OR 2.70, 95% CI 1.76-4.13 and OR 2.44, 95% CI 1.63-3.65 respectively) (Table 2).

**Table 2:** Association of lifetime and twelve-month heavy-drinking with mental illnesses

	**Twelve-month heavy drinking**				**Lifetime heavy drinking**			
**Mental illnesses**^**1**^	**Yes n (%)**	**No n (%)**	**Crude OR (95% CI)**^**2**^	**Adjusted OR (95% CI)**^**3**^	**Yes n (%)**	**No n (%)**	**Crude OR (95% CI)**^**2**^	**Adjusted OR (95% CI)**^**3**^
**Mood disorders**								
Major depressive disorder	46(6.2)	131(1.6)	**3.93(2.44,6.32)**	**3.47(1.98,6.08)**	116(11.6)	294(4.7)	**2.7(1.97,3.7)**	**2.75(1.88,4)**
Dysthmia	5(0.5)	18(0.2)	1.95(0.47,7.98)	2.45(0.32,18.5)	5(0.4)	17(0.3)	1.53(0.37,6.31)	1.92(0.24,15)
Bipolar disorder	13(0.7)	46(0.6)	1.25(0.49,3.15)	1.07(0.36,3.17)	26(2.5)	64(1.0)	**2.64(1.38,5.05)**	**2.17(1.08,4.37)**
Any mood disorder	59(6.9)	175(2.2)	**3.32(2.16,5.11)**	**2.96(1.79,4.92)**	142(14.2)	356(5.6)	**2.79(2.09,3.73)**	**2.78(1.98,3.92)**
**Anxiety disorders**								
Generalized anxiety disorder	5(0.5)	27(0.4)	1.29(0.32,5.12)	1.69(0.28,10.1)	11(0.8)	59(0.9)	0.95(0.37,2.41)	0.9(0.29,2.84)
Obsessive compulsive disorder	15(1.7)	66(1.1)	1.59(0.73,3.44)	1.46(0.57,3.73)	47(4.5)	180(2.7)	**1.69(1.08,2.66)**	1.34(0.78,2.31)
Any anxiety disorder	19(2.1)	89(1.3)	1.59(0.79,3.19)	1.68(0.71,4.02)	56(5.0)	225(3.4)	**1.53(1,2.32)**	1.22(0.74,2.02)
**Alcohol use disorders**								
Alcohol abuse	24(3.6)	3(0.0)	**250(70.9,881)**	**139(36,540)**	178(17.0)	30(0.5)	**45.1(24.3,83.9)**	**50.6(24.5,104)**
Alcohol dependence	18(2.1)	0(−)			34(2.6)	5(0.0)	**101(37.3,273)**	**58.2(19.8,171)**
Alcohol abuse or dependence (alcohol use disorder)	42(5.7)	3(0.0)	**406(120,1372)**	**236(65.2,854)**	212(19.6)	35(0.5)	**50.7(28.2,91.2)**	**53.5(27.2,105)**
**Nicotine dependence**	84(10.0)	262(3.6)	**3.06(2.11,4.43)**	**2.70(1.76,4.13)**	106(9.6)	237(3.4)	**3.00(2.11,4.26)**	**2.44(1.63,3.65)**

OR = Odds Ratios 95% CI = 95% Confidence Intervals. Estimates in bold are statistically significant.

^1^DSM-IV criteria matching diagnoses in the last twelve-months, elicited using the World Mental Health–Composite International Diagnostic Interview.

^2^Simple logistic regression analyses.

^3^Multiple logistic regression analyses; adjusted for adjusted for age (4 age groups), gender, ethnicity (Chinese, Malays, Indians, Others), income level (3 categories), education level (4 categories), marital status (4 categories) and employment status (3 categories).

Among the physical conditions evaluated, inverse associations between lifetime heavy -drinking with hypertension and cancer, and a positive association with respiratory conditions were seen. After multiple adjustments, none of the physical conditions were associated with lifetime heavy drinking except for chronic pain, where there was a significant positive association (OR 1.44, 95% CI 1.07-1.93). The associations seen with hypertension, cancer and respiratory conditions were no longer significant, suggesting that these were confounded by socio-demographic factors (specifically, age) (Table 3). Before adjustment, twelve-month heavy drinking was inversely associated with diabetes, hypertension and cardiovascular conditions, and positively associated with respiratory conditions. None of the physical conditions were associated with twelve-month heavy drinking after adjustment.

**Table 3:** Association of lifetime heavy-drinking with physical disorders

	**Life-time heavy drinking**				**Twelve-month heavy drinking**			
	**Yes n(%)**	**No n(%)**	**Crude ORs (95% CI)**^**1**^	**Adjusted ORs (95% CI)**^**2**^	**Yes n(%)**	**No n(%)**	**Crude ORs (95% CI)**^**1**^	**Adjusted ORs (95% CI)**^**2**^
Diabetes	60(6.8)	587(9.4)	0.70(0.46,1.07)	1.32(0.81,2.16)	37(4.4)	612(9.6)	**0.43(0.25,0.72)**	1.03(0.55,1.93)
Hypertension/High blood pressure	128(14.3)	957(20.8)	**0.64(0.48,0.84)**	1.12(0.78,1.61)	94(12.1)	995(20.8)	**0.52(0.38,0.72)**	1.14(0.77,1.71)
Chronic pain	172(16.9)	797(15.0)	1.15(0.89,1.48)	**1.44(1.07,1.93)**	136(16.4)	837(15.2)	1.10(0.83,1.45)	1.37(0.99,1.89)
Respiratory conditions	142(13.5)	562(8.6)	**1.66(1.25,2.20)**	1.18(0.84,1.66)	117(14.5)	590(8.7)	**1.78(1.32,2.41)**	1.23(0.86,1.76)
Cardiovascular conditions	32(2.6)	198(3.9)	0.66(0.36,1.20)	1.26(0.63,2.51)	17(1.2)	213(4.0)	**0.29(0.13,0.62)**	0.63(0.25,1.61)
Neurological conditions	19(2.4)	179(4.2)	0.56(0.31,1.02)	0.98(0.52,1.85)	13(2.4)	187(4.2)	0.55(0.28,1.09)	1.08(0.52,2.24)
Cancer	5(0.2)	36(0.8)	0.27(0.10,0.78)	0.35(0.10,1.19)	4(0.2)	37(0.8)	**0.30(0.10,0.94)**	0.50(0.14,1.77)
Ulcers and chronic inflamed bowels	25(2.9)	94(1.9)	1.56(0.86,2.82)	1.46(0.69,3.12)	19(2.3)	101(2.0)	1.18(0.60,2.30)	0.933(0.39,2.25)
Any chronic physical condition	400(40.5)	2353(43.1)	0.90(0.74,1.09)	1.19(0.95,1.50)	306(38.0)	2454(43.3)	**0.80(0.65,0.99)**	1.11(0.86,1.43)

OR = Odds Ratios 95% CI = 95% Confidence Intervals. Estimates in bold are statistically significant.

^1^Simple logistic regression analyses.

^2^Multiple logistic regression analyses; adjusted for adjusted for age (4 age groups), gender, ethnicity (Chinese, Malays, Indians, Others), income level (3 categories), education level (4 categories), marital status (4 categories) and employment status (3 categories).

The proportion of participants reporting problems with pain and discomfort was greatest among lifetime heavy drinkers with alcohol use disorder, compared to heavy drinkers without alcohol use disorder and non-heavy drinkers without alcohol use disorder (p = 0.0407). Similarly, there were differences in the proportion with mobility problems in these 3 groups (p = 0.0006), although pairwise comparisons were also not significant. Posthoc comparisons were significant for usual activities, where the proportion of lifetime heavy drinkers with alcohol use disorder who had problems with usual activity was significantly greater than that in non-lifetime heavy drinkers without alcohol use disorder, and for pain/discomfort, where both lifetime heavy drinkers with alcohol use disorder and lifetime heavy drinkers without alcohol use disorder had higher proportions reporting problems with pain and discomfort than among non-heavy drinkers without alcohol use disorder. No other posthoc comparisons were significant (Table 4).

**Table 4:** Association between lifetime and twelve month heavy drinking and EuroQoL health domains, health-related quality of life and work-day loss

	**Lifetime heavy drinking**				**Twelve-month heavy drinking**			
	**A.**	**B.**	**C.**	***P *** **value**^**1**^	**D.**	**E.**	**F.**	***P *** **value**^**1**^
	**Heavy drinkers with alcohol use disorder; n,% reporting moderate/severe problems (SE)**	**Heavy drinkers without alcohol use disorder; n,% reporting moderate/severe problems (SE)**	**Non-heavy-drinkers without alcohol use disorder n,% reporting moderate/severe problems (SE)**		**Heavy drinkers with alcohol use disorder; n,% reporting moderate/severe problems (SE)**	**Heavy drinkers without alcohol use disorder; n,% reporting moderate/severe problems (SE)**	**Non-heavy-drinkers without alcohol use disorder n,% reporting moderate/severe problems (SE)**	
**EuroQol 5D domains**								
Mobility	3.5 (1.6)	3 (1)	3.6 (0.4)	0.8477	3.7 (2.6)	1 (0.4)	3.9 (0.4)	0.0006
Self-care	0.4 (0.4)	0.4 (0.3)	0.6 (0.2)	0.8662	.,.	.,.	0.6 (0.2)	.
Usual activities	5.2 (2.1)	2.7 (1)	2 (0.3)	0.126^a^	4.4 (2.8)	1.3 (0.6)	2.3 (0.3)	0.243
Pain/discomfort	23.4 (4.4)	12.7 (1.8)	15.5 (0.8)	0.0407^b^	12.3 (6.1)	13.6 (1.8)	15.6 (0.8)	0.5537
Anxiety/Depression	10.7 (2.8)	9.3 (1.6)	7.8 (0.6)	0.4072	17.4 (6.7)	7.2 (1.4)	8.2 (0.6)	0.1832
	**A.**	**B.**	**C.**	***P *** **value**^**1**^	**D.**	**E.**	**F.**	***P *** **value**^**1**^
	**Heavy drinkers with alcohol use disorder; mean, (SE)**	**Heavy drinkers without alcohol use disorder; mean, (SE)**	**Non-heavy-drinkers without alcohol use disorder mean, (SE)**		**Heavy drinkers with alcohol use disorder; mean, (SE)**	**Heavy drinkers without alcohol use disorder; mean, (SE)**	**Non-heavy-drinkers without alcohol use disorder mean, (SE)**	
**EuroQol 5D indexed score**								
**All agegroups**	0.918(0.01)	0.956(0.01)	0.951(0.002)	**0.0267**^**c**^	0.930(0.02)	0.960(0.01)	0.950(0.002)	0.4370
18–34	0.916(0.02)	0.963(0.01)	0.971(0.003)	**0.0010**^**d**^	0.913(0.03)	0.963(0.01)	0.969(0.003)	0.0579^h^
35–49	0.912(0.04)	0.951(0.01)	0.954(0.004)	0.5415	0.969(0.03)	0.942(0.01)	0.953(0.004)	0.3237
50–64	0.930(0.03)	0.950(0.02)	0.944(0.01)	0.7128		0.978(0.01)	0.943(0.01)	**0.0078**^**i**^
65 and +	0.962(0.03)	0.917(0.05)	0.914(0.01)	0.5260		0.994(0.01)	0.912(0.01)	0.5131
**EuroQol 5D visual Analogue Scale score**								
**All agegroups**	77.991(1.20)	81.996(0.74)	83.632(0.28)	**<0.001**^**e**^	75.807(2.29)	82.352(0.74)	83.429(0.27)	**<0.0001**^**j**^
18–34	77.362(1.62)	82.926(1.02)	85.307(0.44)	**<0.001**^**f**^	76.370(2.81)	82.897(1.00)	84.981(0.44)	**<0.0001**^**k**^
35–49	77.700(2.0)	81.322(1.28)	84.133(0.47)	**0.0114**^**g**^	74.001(3.78)	81.535(1.27)	83.963(0.46)	**0.0486**^**l**^
50–64	82.774(3.68)	80.212(2.04)	83.308(0.54)	0.6449		81.588(2.29)	83.170(0.53)	0.7741
65 and +	71.427(2.33)	79.504(3.59)	79.119(1.07)	0.1560		81.868(4.56)	78.882(1.04)	0.2687
**Self-reported loss in work days**								
**All agegroups**	0.352(0.10)	0.595(0.14)	0.499(0.06)	0.095	0.407(0.22)	0.385(0.10)	0.524(0.06)	0.515
	0.430(0.16)	0.493(0.14)	0.360(0.05)	0.595	0.408(0.28)	0.403(0.13)	0.387(0.05)	0.986
35–49	0.289(0.17)	0.666(0.27)	0.443(0.09)	0.301	0.404(0.28)	0.479(0.23)	0.457(0.09)	0.418
50–64	0.273(0.23)	0.800(0.65)	0.682(0.16)	0.093	.	0.171(0.10)	0.712(0.16)	**0.022**^**m**^
65 and +	0.130(0.15)	0.767(0.72)	0.597(0.28)	0.551	.	0.035(0.04)	0.640(0.27)	0.716

Estimates in bold are statistically significant.

1 Statistical testing for difference in scores, using multiple linear regression, and adjusted for gender, ethnicity (Chinese, Malays, Indians, Others), income level (3 categories), education level (4 categories), marital status (4 categories) and employment status (3 categories) and any chronic physical conditions.

Significant post hoc tests:

a. A vs. C (p value = 0.0106).

b. A vs. C (p value = 0.0009) & B vs. C (p value = 0.0008).

c. A vs. C (p value = 0.0135).

d. A vs. C (p value = 0.0003).

e. A vs. C (p value < 0.0001) & B vs. C (p value = 0.0190).

f. A vs. C (p value < 0.0001).

g. A vs. C (p value = 0.0158) & B vs. C (p value = 0.0315).

h D vs. F (p value = 0.0419).

i. E vs. F (p value = 0. 0078).

j. D vs. F (p value < 0.0001).

k. D vs. F (p value < 0.0001).

l. D vs. F (p value = 0.0370).

m. E vs. F (p value = 0.0215).

All other two-way comparisons not significant.

Overall, no differences in quality of life as measured on the index derived from the EuroQoL 5D were seen among lifetime heavy drinkers who did not have alcohol use disorder compared to non- heavy drinkers, although lifetime heavy drinkers with alcohol use disorder had a significantly lower score as compared to non-heavy drinkers. No differences were seen with 12-month heavy drinkers, with or without alcohol use disorder, compared to non- heavy drinkers. When stratified by age-group, lifetime and twelve-month heavy drinkers with alcohol use disorder in the 18–34 year age group reported significantly lower quality of life compared to non- heavy drinkers (p = 0.0003, 0.0419 respectively). Twelve-month heavy drinkers without alcohol use disorder aged 50–64 reported higher quality of life than older non- heavy drinkers in the 50–64 year age group, (p = 0.0078) (Table 4).

Lifetime heavy drinkers with alcohol use disorder and without alcohol use disorder reported poorer quality of life compared to non- heavy drinkers without alcohol use disorder (p < 0.0001 and 0.019, respectively) on the VAS. Among 12-month heavy -drinkers, significantly lower quality of life was reported only among those with alcohol use disorder (p = <0.0001). Lifetime and 12-month heavy drinkers with alcohol use disorder reported significantly lower quality of life in both the 18–34 year (p < 0.0001 and 0.0158 respectively) and the 35–49 year (p < 0.0001 and 0.037 respectively) age groups. No significant difference in quality of life was reported by lifetime and 12 month heavy drinkers without alcohol use disorder, except for lifetime heavy drinkers in the 35–49 year age group, which reported a significantly lower quality of life (p = 0.0315). No clear differences in self-reported loss in work days were seen, with or without stratification by age, except for 12 month heavy drinkers without alcohol use disorder in the 50–64 year age group, where they reported fewer days lost (p = 0.022) (Table 4).

## Discussion

In this study of a representative population of adult Singaporeans and Singapore permanent residents, we found that 12.6% of all adult Singapore residents reported heavy drinking in the past 12 months, and 15.9% reported lifetime heavy-drinking. There were strong socio-demographic differences between heavy-drinkers and non-drinkers, with heavy-drinkers being more likely to be men, younger, single, and earning higher income. Malays were much less likely to be heavy drinkers compared to the Chinese. Heavy drinking was positively associated with major depression, the presence of any mood disorder, and chronic pain. It was also strongly associated with alcohol dependence and alcohol abuse, and nicotine dependence. Overall, heavy drinkers reported lower quality of life compared to non- heavy drinkers on the EuroQoL indexed score and Visual Analogue Scale.

Data from the National Health Survey in 2004 previously suggested a binge-drinking prevalence of about 10% [13]. Our results of 12.6% for heavy drinking are consistent with the data obtained from 2004. Additionally, both studies identified the young, males, and people with higher incomes as being more likely to drink heavily, while Malays were less likely to do so [13]. The strong ethnic differences in heavy-drinking rates are likely to be due to the role of culture and religion as a protective factor. Malays in Singapore are almost all Muslims, for whom there is a religious prohibition on alcohol consumption. Further analyses of our data show that the proportion of heavy drinkers (both over twelve months or lifetime) in Malays who drink was similar to or higher than the proportion in Chinese who drink (not shown in tables), in contrast to the overall prevalence of heavy drinking, which is much lower among Malays. This suggests that the effect of ethnicity on heavy drinking works via reducing initiation of drinking rather than drinking volume. This is consistent with the hypothesis that the protective effect of ethnicity on heavy drinking is mediated through religion, since Islam prohibits alcohol consumption. We also note a very high prevalence of alcohol consumption among people of “Others” ethnicities, with an odds of heavy drinking of about 7 times higher than the Chinese. This increase is statistically significant. In Singapore, the majority of people classified as “Others” are people of Eurasian or Caucasian origins, although other ethnicities such as Thai, or Japanese are also classified in this group. Further research is needed to evaluate which ethnicities within this group are drinking more, and to understand the context of the heavy alcohol consumption, so that appropriate intervention programmes can be designed and implemented.

The prevalence of heavy drinking is much lower than those reported in non-Asian countries [9,10]. Heavy drinking in Singapore is primarily a condition of the young, as has been reported in other studies [9,10]. Our findings with regard to income and marital status are consistent with findings reported previously [9,10,19]. We did not detect an inverse association with education, which has been reported elsewhere [19].

Alcohol use in Singapore in the past was much lower compared to Western and some Asian countries, with low rates seen particularly amongst Malays and women [13]. However, both published studies and anecdotal evidence suggest that alcohol use is becoming accepted as a social norm in Singapore [13]. Alcohol use disorder has increased in recent years, especially among the young [12]. Alcohol use and abuse control policies in Singapore depend on a variety of measures, including heavy taxation on alcohol products [20], legislation such as restrictions on sale of alcohol to minors and anti-drink-driving laws, and promotional campaigns to curb misuse of alcohol by the Health Promotion Board and the Traffic Police [21].

We found an association of heavy drinking with mental illnesses, in particular major depression, also consistent with findings from other studies [2,22,23]. The strong association with alcohol abuse and use is not unexpected. However, the vast majority of heavy drinkers do not have alcohol use disorders, and alcohol use disorders do not adequately predict heavy drinking. The association of heavy drinking with depression, and to a lesser extent, with anxiety disorders, is consistent with the higher prevalence of unhealthy lifestyle behaviour (including poorer diet, lower levels of physical activity, higher smoking prevalence) that has been reported among people with psychiatric disorders [24,25]. Heavy drinking was also positively and strongly associated with nicotine dependence, again consistent with results from other studies [26]. In our data, more than a quarter of people who fulfilled criteria for major depressive disorder reported heavy drinking in the last 12 months. This suggests that doctors managing patients with depression may need to assess for and manage unsafe alcohol use in their patients even in the absence of alcohol abuse or alcohol dependence. There is some evidence that reducing alcohol consumption might improve depressive symptoms [2].

We note a significant positive association between chronic pain and lifetime heavy drinking, and a borderline significant finding with twelve-month heavy drinking. Further, heavy drinkers with or without alcohol use disorder reported poorer scores on the pain/discomfort dimension on the EuroQoL 5D compared to non-heavy drinkers. A possible explanation for this association is that this relationship could be mediated through mental disorders such as major depression: we observe a positive association between heavy drinking and depression in this paper, and previous analyses of our dataset have shown that the prevalence of mental disorders including depression is higher among those with chronic pain [27]. Another explanation for these associations may be that the higher prevalence reflects attempts by persons living with depression or chronic pain to alleviate their symptoms through the use of alcohol.

Our results show that the greatest declines to quality of life and to work-day loss relate to the presence of mental illnesses, and do not suggest that heavy drinking in the absence of alcohol use disorder is associated with significant loss of quality of life, nor with loss in work-days. Other studies have reported changes in quality of life associated with binge drinking [28,29], and a recent study in Norway has suggested that binge drinking is associated with absenteeism in the workforce [30]. This discrepancy could be due to a ceiling effect in the instruments used [31], with a relative lack of discrimination among otherwise well individuals. We did detect differences in quality of life among younger participants who drank heavily, compared to those who did not, on the VAS but not using the index score. These results are consistent with findings from other countries (28,29), and suggest that for younger, otherwise healthy populations, the VAS may be more discriminatory than the index scores.

In the older age-groups, higher quality of life was unexpectedly reported among heavy drinkers without alcohol use disorder compared to non-heavy drinkers. This may reflect reverse causation where healthy individuals are more likely than persons with physical illnesses to continue drinking, or it may be that there is no decline in quality of life associated with heavy drinking for the majority of drinkers, who may find the social interaction associated with drinking a strong promoter of emotional well-being. Interestingly, a recent study in the US also reported that heavy drinkers had higher measures of positive health in some domains [32].

There are several limitations in our study. We used a cross-sectional design, and while our data give nationally representative figures, we were unable to establish the causality of associations that we have found. Our contact rate of 67.5% and response rate of 75.9% are reasonable and consistent with response rates obtained in population-based surveys conducted elsewhere. However, contact and response rates of heavy drinkers may differ from non-heavy drinkers, and this differential response rate may bias our prevalence estimates. *A priori*, we assume that heavy drinkers are likely to have lower contact and response rates. If so, our prevalence estimates would represent a lower limit, and the true prevalence may be higher than estimated.

We used an operational definition of heavy drinking as a consumption of 4 or 5 drinks (depending on gender) in a single day, in contrast to the traditional binge drinking definition of 4 or 5 units over a single drinking episode. Our results therefore does not measure 12-month binge drinking rates, and direct comparisons with binge drinking rates cited in other studies are not possible. Because binge drinkers are heavy drinkers, but heavy drinkers need not be binge drinkers, our prevalence estimate may be an overestimate of the 12-month binge drinking rate, and serve as an upper estimate of the true binge drinking rate. The definition for 12- month heavy drinking (largest number of drinks in a single day) was different from lifetime heavy drinking (usual number of drinks). The lifetime heavy drinking estimate will underestimate the true prevalence of lifetime binge drinking. We believe 12-month heavy drinking is a better proxy for binge drinking than the lifetime one.

Finally, we used face-to-face interviews for this study. Participants may not have reported accurate consumption rates because of their perceptions of the social acceptability of alcohol consumption. This may be so especially amongst females or Malays, where societal expectations are that they do not drink. Conversely, young males may have over-reported their consumption patterns. We have tried to reduce this misclassification bias by training our interviewers to administer the interviews in a sensitive and neutral manner, and using standard questionnaires.

## Conclusions

In summary, despite low reported rates of alcohol abuse and alcohol dependence, we find a relatively high prevalence of 12-month heavy drinking of 12.6%, and lifetime heavy drinking of 15.9%. Although these rates are lower compared to other countries, it is important to continue to monitor alcohol consumption trends. Efforts to improve awareness among the young of the ill effects of heavy drinking, and to mitigate harms associated with heavy drinking and intoxication also need to be continued.

## Competing interests

All authors declare that they have no conflicts of interests.

## Authors’ contributions

SAC, MS and JV designed and conducted the study. W-YL conducted literature searches and provided summaries of previous research studies. EA, VYFH and W-Y Lim conducted the statistical analysis. W-Y Lim drafted the manuscript and all authors have contributed to and have approved the submission of this manuscript.

## Pre-publication history

The pre-publication history for this paper can be accessed here:

http://www.biomedcentral.com/1471-2458/13/992/prepub
